# Methicillin-Resistant *Staphylococcus schleiferi* Subspecies *coagulans* Infection in a Patient With Hepatocellular Carcinoma

**DOI:** 10.1177/2324709616671148

**Published:** 2016-09-26

**Authors:** Thein Swe, Akari Thein Naing, AAMA Baqui, Ratesh Khillan

**Affiliations:** 1 Department of Internal Medicine, Interfaith Medical Center, New York, NY, USA; 2 Department of Microbiology and Pathology, Interfaith Medical Center, New York, NY, USA; 3 Division of Oncology, Interfaith Medical Center, New York, NY, USA

**Keywords:** hepatocellular carcinoma, *Staphylococcus schleiferi* subspecies *coagulans*, methicillin resistant

## Abstract

To our knowledge and literature search, *Staphylococcus schleiferi* subspecies *coagulans* infection in human beings has rarely been described in the medical literature. Furthermore, we believe that this is a first detailed case report of methicillin-resistant *Staphylococcus schleiferi* subspecies *coagulans* infection in a patient with hepatocellular carcinoma. Because of the possible association of *Staphylococcus schleiferi* infection and immunosuppression, any isolates of this bacterium in human beings should be presumed to be pathogenic, unless proven otherwise.

## Introduction

*Staphylococcus schleiferi* is one of the Staphylococcus species that can cause skin and ear infections in dogs. It was first described in 1988.^1^ Almost all strains of *S schleiferi* produce lipase, esterase, and β-hemolysin as markers of virulence. *S schleiferi* are susceptible to novobiocin and produce a heat-stable nuclease. They are coagulase-negative organisms and can differentiate from Staphylococcus aureus by production of different nuclease with lack of pigmentation. *S schleiferi* has 2 distinct subspecies, which are known as *S schleiferi* subsp schleiferi and *S schleiferi* subsp *coagulans*. They can be distinguished by activity of tube coagulase and urease. *S schleiferi* subsp *schleiferi* is tube coagulase and urease negative, whereas *S schleiferi* subsp *coagulans* is tube coagulase and urease positive.^2^

*S schleiferi* can become clinically important diseases in human beings as wound or surgical site infections,^3^endocarditis,^2^ pediatrics meningitis,^4^ brain empyema, and intravascular device-related bacteremia including pacemaker.^5^ *S schleiferi* subspecies *coagulans* infection in humans has rarely been reported, and we believe this case report is the first detailed description about *S schleiferi* subsp *coagulans* infection in a patient with hepatocellular carcinoma.

## Case Presentation

A 66-year-old male patient was admitted to the hospital because of altered mental status for 1 day. Vital signs included temperature 94°F (34.4°C), pulse rate 101 beats/minutes, respiratory rate 23 breaths/minutes, blood pressure 130/73 mm Hg, and oxygen saturation 98%. Physical examination showed a lethargic and disoriented patient with respiratory distress. Respiratory examination revealed fast breathing with equal breath sounds without crepitations or rhonchi. Cardiovascular examination showed tachycardia, normal first and second heart sounds without murmur. Abdomen was distended with free fluid.

On the day of admission, laboratory tests showed white blood cells (WBC) 19.2 × 10^9^/L, neutrophils percentage 73.8%, absolute neutrophil count 14 2000/µL, bilirubin 0.5 mg/dL, aspartate transaminase 56 IU/L, alanine transaminase 46 IU/L, alkaline phosphatase 66 IU/L, ammonia 34 µmol/L, tumor marker α-fetoprotein level 2500 ng/mL (normal = 0-8.3), and lactic acid 2.6 mmol/L. Serum coagulation profile, electrolytes, amylase, and lipase were within normal limits. Urinalysis revealed WBC 5 cells/high power field, many bacteria, negative nitrate, and negative leucocyte esterase. Chest X-ray was unremarkable.

Subsequently, the patient developed hypotension 80/60 mm Hg. The patient was intubated and put on mechanical ventilation. Two sets of blood cultures were drawn from veins of hands of the patient at different times, and he was given normal saline and empiric antibiotics intravenously. The patient is a chronic alcoholic and has past medical history of chronic hepatitis C (not sure whether it was treated or not). Computed tomography scan of abdomen and pelvic showed hemoperitoneum in the abdomen and pelvis and heterogeneous mass-like density abutting the gallbladder and lower right hepatic lobe.

After 48 hours, one set of blood culture (which contains 2 bottles: one aerobic bottle and one anaerobic bottle)grew methicillin-resistant *S schleiferi* subspecies *coagulans*. It was found in one medium at 48 hours with minimum inhibitory concentration level of oxacillin of 1 µg/mL. The bacteria were sensitive to ciprofloxacin, daptomycin, gatifloxacin, gentamicin, levofloxacin, linezolid, rifampin, synercid, tetracycline, trimethoprim/sulfamethoxazole, and vancomycin. However, it was resistant to ampicillin, ampicillin/sulbactam, cefazolin, clindamycin, erythromycin, penicillin, and oxacillin. Urine culture and transtracheal aspiration cultures were negative. The patient was treated with vancomycin 1 g twice daily intravenously for 7 days. On day 5 of hospital stay, WBC counts were normalized, 10 × 10^9^/L, with absolute neutrophils count of 7700/µL. Later, the patient was diagnosed with ruptured hepatocellular carcinoma. The patient underwent laproscopic resection of liver tumor and biopsy revealed ruptured multinodular hepatocellular carcinoma. Repeated blood cultures were negative. Patient mental status, clinical condition, and vital signs improved in a few days and he was discharged. He was recommended to follow-up with the clinic; however, he was lost to follow-up.

## Discussion

*Staphylococcus schleiferi* is often mistaken with *S aureus* because both organisms have heat-stable DNase and clumping factor. The subspecies *schleiferi* can produce a pseudocoagulase although protease inhibitors and anticoagulants can often inhibit clotting activity and thus it is mostly assumed as coagulase negative.^6,7^*S schleiferi* subsp *coagulans* are gram-positive nonmotile cocci that are facultatively anaerobic. The strains produce free coagulase (test tube coagulase test with rabbit plasma). However, they fail to produce fixed coagulase, which is clumping factor with human plasma.^8^*S schleiferi* subsp *coagulans* can be differentiated from other coagulase-positive species with its acetoin production, negative hyaluronidase activity, and lack of acid production from maltose.^8^

Infection with *S Schleiferi* was seen as uropathogenic organisms in 2 elderly and 1 pediatric hospitalized patients.^9^ *S schleiferi* subsp *coagulans* infection in human is a rare condition. *Leung* et al described a case with endocarditis,^2^and Thibodeau et al reported a patient with left ventricular assist device infection awaiting heart transplantation.^6^

A study from a tertiary care center in Spain revealed that *S schleiferi* infections were more common in males and more than half of the patients had evidence of immunosuppression, mainly malignant neoplasms.^*10*^ Furthermore, there was one case report about endocarditis and metastatic infection in an immune comprised host who recovered with conventional treatment.^11^Our patient also had hepatocellular carcinoma, and *S schleiferi* infection in human may have possible association with immunosuppression and neoplasm.

In conclusion, this is the first detailed case description about methicillin-resistant *Staphylococcus schleiferi* subspecies *coagulans* infection in a male patient with hepatocellular carcinoma. A high index of suspicion is required between the possible association of *S schleiferi* infection in human with immunosuppression, mainly neoplasm becauseany isolates of this bacterium in human beings should be presumed to be pathogenic, unless proven otherwise.
